# Alpha-Lipoic Acid Attenuates Cerebral Ischemia and Reperfusion Injury via Insulin Receptor and PI3K/Akt-Dependent Inhibition of NADPH Oxidase

**DOI:** 10.1155/2015/903186

**Published:** 2015-07-29

**Authors:** Yinhua Dong, Hongxin Wang, Zefeng Chen

**Affiliations:** Department of Neurology, The Affiliated Fourth Centre Hospital of Tianjin Medical University, Tianjin 300140, China

## Abstract

Alpha-lipoic acid (ALA) has various pharmacological effects such as antioxidative, anti-inflammatory, and antiapoptotic properties. In the present study, administration of ALA (40 mg/kg, i.p.) for 3 days resulted in a significant decrease in neuronal deficit score and infarct volume and a significant increase in grip time and latency time in Morris water maze at 48 h after middle cerebral artery occlusion and reperfusion (MCAO/R) in rats. ALA also reduced the increased TUNEL-positive cells rate and the enhanced caspase-3 activity induced by MCAO/R. However, the underlying mechanisms remain poorly understood. In this study, we found that ALA could activate insulin receptor and PI3K/Akt signaling pathways, inhibit the expression and activity of NADPH oxidase, and subsequently suppress the generation of superoxide and the augment of oxidative stress indicators including MDA, protein carbonylation, and 8-OHdG. In conclusion, ALA attenuates cerebral ischemia and reperfusion injury via insulin receptor and PI3K/Akt-dependent inhibition of NADPH oxidase.

## 1. Introduction

Ischemic stroke is a major cause of disability and the second cause of death worldwide [1]. Despite considerable progress in the understanding of the pathophysiology of ischemic stroke in recent years, therapeutic options have until now been limited. The only approved drug for ischemic stroke is recombinant tissue plasminogen activator [2]. Nevertheless, even though blood flow is restored timely, reperfusion can paradoxically exacerbate brain injury because of neuronal oxidative stress.

Mechanistically, oxidative stress resulting from the overproduction of reactive oxygen species (ROS) is implicated in the pathophysiology of cerebral ischemia and reperfusion (CIR) injury. Based on this hypothesis, ROS-scavenging antioxidants have been speculated to be neuroprotective against ischemic stroke. However, numerous ROS-scavenging antioxidants have shown disappointing results in clinical trials. Inhibiting ROS generation is a novel therapeutic approach to suppress oxidative stress at its root [3]. However, the sources of ROS in CIR injury are largely unknown.

Among the sources of ROS, only NADPH oxidase can primarily produce ROS as the primary production in CIR injury [4]. Previous study has demonstrated that NOX KO mice showed less brain infarction compared with wild-type (WT) mice after MCAO/R [5]. Therefore, NADPH oxidase is a promising therapeutic target for ischemic stroke. Moreover, the activity of NADPH oxidase is reportedly regulated by several signaling pathways such as insulin receptor, PI3K/Akt, and MAPKs pathways [6–8].

Recent studies have demonstrated that endogenous antioxidants such as superoxide dismutase, glutathione, and alpha-lipoic acid (ALA) have neuroprotective effects [9–11]. Several studies have indicated that ALA possesses numerous biological effects including antioxidative, anti-inflammatory, and antiapoptotic properties [12, 13]. ALA is reported to provide neuroprotection against CIR injury via inhibiting oxidative stress [14, 15]. However, whether the neuroprotective effects of ALA against oxidative stress are due to inhibiting NADPH oxidase remains to be investigated. ALA is reported to be a directly binding activator of the insulin receptor [16]; whether activation of insulin receptor induced by ALA is responsible for its neuroprotection against CIR injury remains to be clarified.

In the present study, a rat model of middle cerebral artery occlusion/reperfusion (MCAO/R) was used to investigate the neuroprotective effects of ALA. We demonstrated that ALA attenuated CIR injury via insulin receptor/Akt-dependent inhibition of NADPH oxidase.

## 2. Methods

### 2.1. Materials

ALA, paraformaldehyde, and 2,3,5-triphenyltetrazolium chloride (TTC) were purchased from Sigma-Aldrich (MO, USA). Tissue protein extraction kits, the bicinchoninic acid assay (BCA) kits, the primary antibodies, and the respective secondary antibodies were purchased from Santa Cruz Biotechnology (CA, USA). The malondialdehyde (MDA) detection kits and caspase-3 activity assay kits were obtained from Nanjing Jiancheng Bioengineering Institute (Nanjing, China). The ELISA kits for protein carbonyl and 8-hydroxydeoxyguanosine (8-OHdG) were purchased from Cell Biolabs (CA, USA).

### 2.2. ALA Solution Preparation

ALA (80 mg/mL) was dissolved in 10% of ethanol and sterilely filtered. ALA solutions were prepared immediately before use.

### 2.3. Animals

All animal protocols were performed in compliance with the National Institute of Health Guide for the Care and Use of Laboratory Animals and approved by the Animal Ethics Committee of Tianjin Medical University. All efforts were made to minimize the injuries experienced by the animals as well as the number of animals used. In this study, adult male Sprague-Dawley rats (Vital River Laboratories, Beijing, China) weighing 280–300 g were used. The rats were housed in a temperature (22°C ± 1°C) and humidity (60% ± 10%) controlled environment with a 12 : 12 h light-dark cycle and given free access to food and water. The rats (*n* = 60) were randomly assigned to 3 groups, that is, the sham group (*n* = 20), the MCAO/R group (*n* = 20), and the ALA + MCAO/R group (*n* = 20). For the ALA + MCAO/R group, ALA (40 mg/kg) was daily administered intraperitoneally (i.p.) to rats for 3 days. For the sham and MCAO/R groups, the rats were given the same amount of ethanol.

### 2.4. Cerebral MCAO/R Surgery

Cerebral MCAO/R was performed in rats following a previously described method [17] with slight modification by the same skilled investigator. Briefly, the surgical procedure was performed under sterile conditions with autoclaved surgical instruments and materials. The rats were anesthetized using a face mask with 3.5% halothane and maintained with 1.0–2.0% halothane in 70% N_2_O and 30% O_2_. The common carotid artery, internal carotid artery (ICA), and external carotid artery (ECA) were carefully exposed. A 4-0 suture (Ethicon Inc., NJ, USA) was inserted into ICA through ECA and was gently pushed further to occlude the middle cerebral artery (MCA). Two h after MCA occlusion, reperfusion was achieved by careful suture removal. The incision was sewed after reperfusion was confirmed using a laser Doppler flowmeter. The rats in the sham group underwent the same surgical procedure without occlusion. To relieve pain and discomfort in the postoperative period, topical lidocaine gel was applied on the wound of rats. The cranial temperature and rectal temperature were maintained at 37 ± 0.5°C using a feedback-regulated water-heating system. Regional cerebral blood flow (rCBF), mean arterial blood pressure (MABP), pH, arterial blood gases, and blood glucose levels were monitored throughout surgery.

### 2.5. Neurological Deficit Score

At 48 h after reperfusion, the neurological deficit (*n* = 20 for each group) was scored in a blinded fashion with regard to the experimental groups according to a previously described method [18] with slight modification, 0, no deficit; 1, mild forelimb weakness; 2, severe forelimb weakness; 3, compulsory circling; 4, no spontaneous walking with depressed consciousness level; and 5, death.

### 2.6. Grip Test

At 48 h after reperfusion, forepaw grip test (*n* = 20 for each group) was performed to evaluate force and fatigability of rats by two investigators who are blinded to the experimental groups. A metallic rope was stretched horizontally 50 cm over a cotton pad. The rats were suspended on the rope by forepaws and the grip time was recorded.

### 2.7. Rota-Rod

The motor and coordination of rats was detected using Rota-rod. Before surgery, the rats (*n* = 20 for each group) were familiarized with the apparatus. At 48 h after reperfusion, the rats were placed on the rotating shaft and the rotate speed was increased from 4 to 40 r/min. The latency time was recorded automatically by the apparatus when the rat fell off the rotating shaft.

### 2.8. Cerebral Infarct Volume

Cerebral infarct volume was determined by TTC staining. At 48 h after reperfusion, the rats (*n* = 10 for each group) were decapitated. The brains were removed quickly and then frozen at −80°C for 15 min and then sliced into consecutive 2 mm coronal sections. The brain slices were incubated with 2% TTC at 37°C for 30 min and then incubated with 10% paraformaldehyde overnight. The images were captured and the corrected infarct volume was calculated according to a previously described method [19].

### 2.9. Terminal Deoxynucleotidyl Transferase dUTP Nick End Labeling (TUNEL)

Apoptotic neurons were detected by TUNEL using ApopTag Fluorescein In Situ Apoptosis Detection Kit (Millipore, MA, USA) in compliance with the manufacturer's protocol. At 48 h after reperfusion, the rats (*n* = 5 for each group) were decapitated. The brains were removed rapidly, embedded, and sliced in a cryostat (20 *μ*m thickness, 100 *μ*m intervals). After deparaffinization and rehydration, the brain slices were incubated with proteinase K (20 *μ*g/mL) for 15 min and incubated in 3% H_2_O_2_ for 5 min. The brain slices were then incubated with working-strength TdT enzyme at 37°C for 1 h in a humidified chamber. After rinsing in the stop/wash buffer, the brain slices were incubated with the working-strength antidigoxigenin conjugate for 30 min. The nucleus was counterstained by 4′,6-diamidino-2-phenylindole (DAPI, 0.1 *μ*g/mL). The images were captured using a fluorescence microscope (Leica, Germany).

### 2.10. Western Blot

The whole cell lysates of cortical samples (*n* = 5 for each group) were obtained using tissue protein extraction kits supplemented with protease inhibitors (Roche, IN, USA). The total protein concentration was determined using BCA kits. An equal amount of protein was separated by electrophoresis in 10% sodium dodecyl sulfate polyacrylamide gels and then transferred to nitrocellulose membranes. After blocking with 5% nonfat milk, the blots were incubated with the primary antibodies overnight at 4°C. After washing with TBST, the blots were then incubated with the respective secondary antibodies for 2 h and developed by chemiluminescence. Relative optical densities of the blots were quantified by densitometry.

### 2.11. NADPH Oxidase Activity Assay

NADPH oxidase activity was detected by superoxide dismutase-inhibitable cytochrome c reduction assay [20]. Briefly, tissue homogenate (*n* = 5 per group) was distributed in 96-well plates (200 *μ*g/well). NADPH (100 *μ*mol/L) and cytochrome c (500 *μ*mol/L) were added in the presence or absence of SOD (200 U/mL) and then incubated for 30 min. The absorbance was measured at 550 nm using a microplate reader. NADPH oxidase activity was expressed as the reduced cytochrome c per mg protein per min as relative absorbance units (RAU).

### 2.12. Detection of Superoxide Production

Lucigenin-enhanced chemiluminescence was employed to measure the production of superoxide [21]. Briefly, cortical homogenate (*n* = 5 per group) was distributed in white 96-well plates (100 *μ*g/well). Lucigenin (5 *μ*mol/L) and NADPH (100 *μ*mol/L) were separately added immediately before reading using a microplate luminometer. The level of superoxide production was expressed as the percentage of the sham group.

### 2.13. Detection of Oxidative Stress and Apoptosis Related Parameters

The total protein concentration of the cortical homogenate (*n* = 5 for each group) was determined using BCA kits. The levels of MDA and caspase-3 activity were detected using respective assay kits. The levels of protein carbonyl and 8-OHdG were determined by respective ELISA kits. The levels of caspase-3 activity, protein carbonyl, and 8-OHdG were expressed as the percentage of the sham group.

### 2.14. Statistical Analysis

Data were expressed as means ± standard error of the mean (SEM). The results were analyzed by one-way ANOVA followed by Tukey's test or two-way ANOVA followed by Bonferroni's multiple comparison test. *P* < 0.05 was considered statistically significant.

## 3. Results

### 3.1. Physiologic Parameters

As shown in supplementary Table 1 in Supplementary Material available online at http://dx.doi.org/10.1155/2015/903186, before occlusion, during occlusion, and after reperfusion, no significant intergroup difference was noted in the physiologic parameters including cranial temperature, rectal temperature, MABP, glucose, pH, PO_2_, and PCO_2_ (*P* > 0.05) and no significant difference was observed in rCBF between MCAO/R and ALA + MCAO/R groups.

### 3.2. The Cytotoxicity and Dose Selection of ALA

To explore the cytotoxicity of ALA, ALA (20, 40, and 80 mg/kg, i.p.) was administered to rats for 3 days. As seen in supplementary Figure 1, ALA (20, 40, and 80 mg/kg) did not affect the caspase-3 activity, NADPH oxidase activity, NADPH oxidase-derived superoxide production, and the levels of MDA, protein carbonyl, and 8-OHdG in cortex of rats. To investigate the protective effects of ALA against CIR injury, ALA (20, 40, and 80 mg/kg, i.p.) was administered to rats for 3 days before surgery. As seen in supplementary Figure 2, at the dose of 40 mg/kg, ALA provided the maximum neuroprotection against MCAO/R-induced neurologic deficits and cerebral infarction. Moreover, neurologic deficits and cerebral infarction induced by MCAO/R were attenuated by pretreatment but not posttreatment with ALA.

### 3.3. ALA Ameliorated MCAO/R-Induced Neurological Deficits

**Figure 1:**
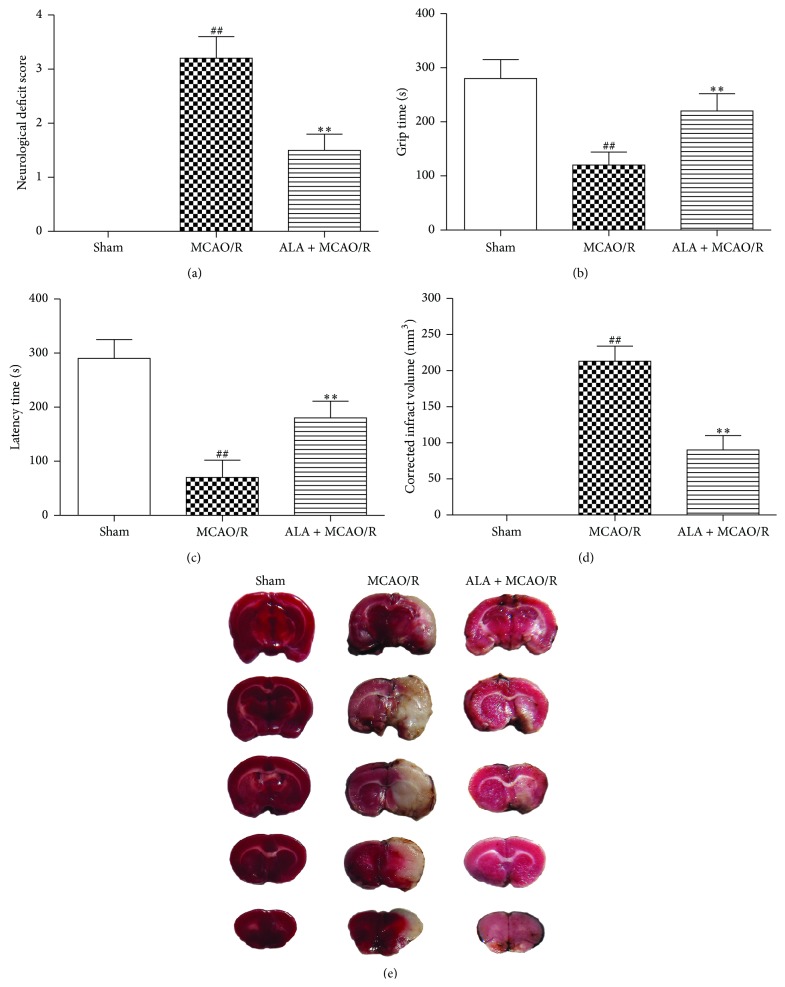
ALA improved neurological functions and attenuated MCAO/R-induced infarct volume. (a) ALA attenuated MCAO/R-induced neurological deficit. (b) ALA ameliorated MCAO/R-induced reduction of grip time. (c) ALA attenuated MCAO/R-induced decrease in latency time in Morris water maze. (d) Quantitative analysis of cerebral infarct volume. (e) ALA inhibited MCAO/R-induced cerebral infarct. For the ALA + MCAO/R group, ALA (40 mg/kg, i.p.) was daily administered to rats for 3 days. For the sham and MCAO/R groups, the rats were given the same amount of ethanol. Data were expressed as means ± SEM. ^##^ *P* < 0.01 compared with the sham group. ^*∗∗*^ *P* < 0.01 compared with the MCAO/R group.

Neurological function was evaluated by neurological deficit score, grip time test, and Rota-rod test at 48 h after reperfusion. Compared to the sham group, MCAO/R-treated rats displayed a significant increase in neurological deficit score (Figure 1(a), *P* < 0.01), a remarkable decrease in grip time (Figure 1(b), *P* < 0.01), and a striking reduction in Rota-rod latency time (Figure 1(c), *P* < 0.01). These results indicated that MCAO/R induced a significant neurological deficit in rats. However, ALA significantly reversed these MCAO/R-induced changes in neurological function (Figures 1(a), 1(b), and 1(c), *P* < 0.01).

### 3.4. ALA Attenuated MCAO/R-Induced Cerebral Infarction

Cerebral infarction was determined by TTC staining. The rats in the sham group displayed no cerebral infarction. However, a significant increase in cerebral infarct volume was observed in MCAO/R-treated rats (Figures 1(d) and 1(e), *P* < 0.01). Conversely, ALA remarkably reduced the augmented cerebral infarct volume induced by MCAO/R (Figures 1(d) and 1(e), *P* < 0.01).

### 3.5. ALA Reduced MCAO/R-Induced Neuronal Apoptosis

**Figure 2:**
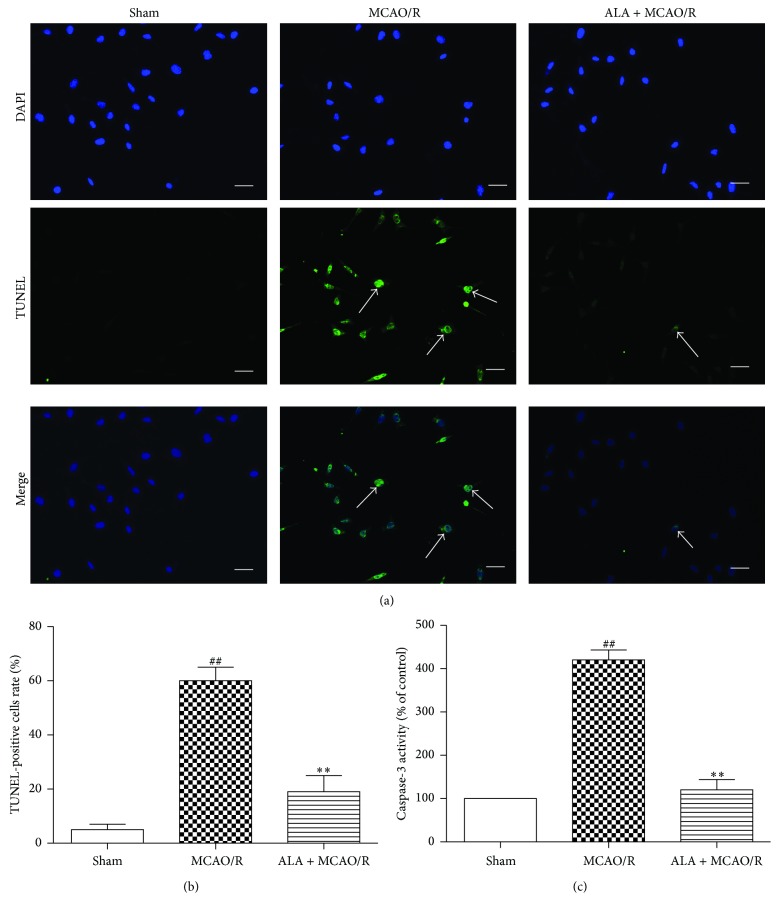
ALA attenuated MCAO/R-induced neuronal apoptosis. (a) DNA fragmentation in apoptotic neuronal cells was detected by TUNEL; arrows represent TUNEL-positive cells; bar = 50 *μ*m. (c) Quantitative analysis of TUNEL-positive cells rate. For the ALA + MCAO/R group, ALA (40 mg/kg, i.p.) was daily administered to rats for 3 days. For the sham and MCAO/R groups, the rats were given the same amount of ethanol. Data were expressed as means ± SEM. ^##^ *P* < 0.01 compared with the sham group. ^*∗∗*^ *P* < 0.01 compared with the MCAO/R group.

DNA fragmentation and caspase-3 activity were detected to investigate whether ALA could affect MCAO/R-induced neuronal apoptosis. The number of TUNEL-positive cells was significantly higher in the cortex of MCAO/R-treated rats at 48 h after reperfusion compared with the sham group (Figures 2(a) and 2(b), *P* < 0.01). Consistent with these observations, caspase-3 activity in the cortex of MCAO/R-treated rats was also significantly enhanced compared to the sham group (Figure 2(c), *P* < 0.01). In contrast, ALA considerably inhibited MCAO/R-induced neuronal apoptosis (Figure 2, *P* < 0.01).

### 3.6. ALA Inhibited NADPH Oxidase through Activation of Insulin Receptor and PI3K/Akt Signaling Pathway

**Figure 3:**
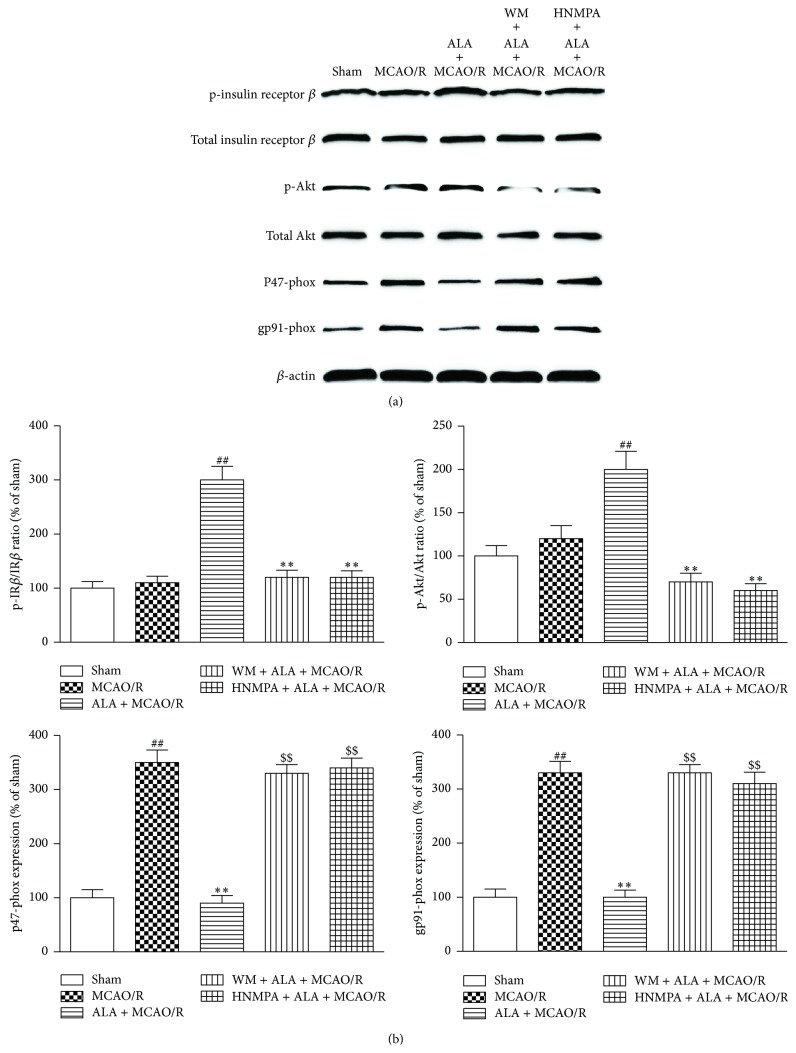
ALA inhibited NADPH oxidase through activation of insulin receptor and PI3K/Akt signaling. (a) The expression of p-insulin receptor, total insulin receptor, p-Akt, total Akt, p47-phox, and gp91-phox. (b) Quantitative analysis of the expression of proteins. For the ALA + MCAO/R group, ALA (40 mg/kg, i.p.) was daily administered to rats for 3 days. For the sham and MCAO/R groups, the rats were given the same amount of ethanol. Data were expressed as means ± SEM. ^##^ *P* < 0.01 compared with the sham group. ^*∗∗*^ *P* < 0.01 compared with the MCAO/R group. ^$$^ *P* < 0.01 compared with the ALA + MCAO/R group.

p47-phox and gp91-phox, two subunits of NADPH oxidase, were significantly higher in the cortex of MCAO/R-treated rats than that of the sham group. However, the increased expression of p47-phox and gp91-phox induced by MCAO/R was effectively inhibited by ALA. Interestingly, ALA could induce the phosphorylation of Akt. Importantly, all these effects mediated by ALA could be abolished by coadministration with PI3K specific inhibitor wortmannin (WM, 0.3 mg/kg, i.p.) or insulin receptor inhibitor HNMPA-(AM)_3_ (5 mg/kg, i.p.) for 3 days (Figures 3(a) and 3(b), *P* < 0.01). Moreover, ALA could induce the phosphorylation of insulin receptor, which was abrogated by HNMPA-(AM)_3_ but not WM. These results suggested that ALA inhibited NADPH oxidase through activation of insulin receptor and PI3K/Akt signaling pathway.

### 3.7. ALA Suppressed MCAO/R-Induced Oxidative Stress

**Figure 4:**
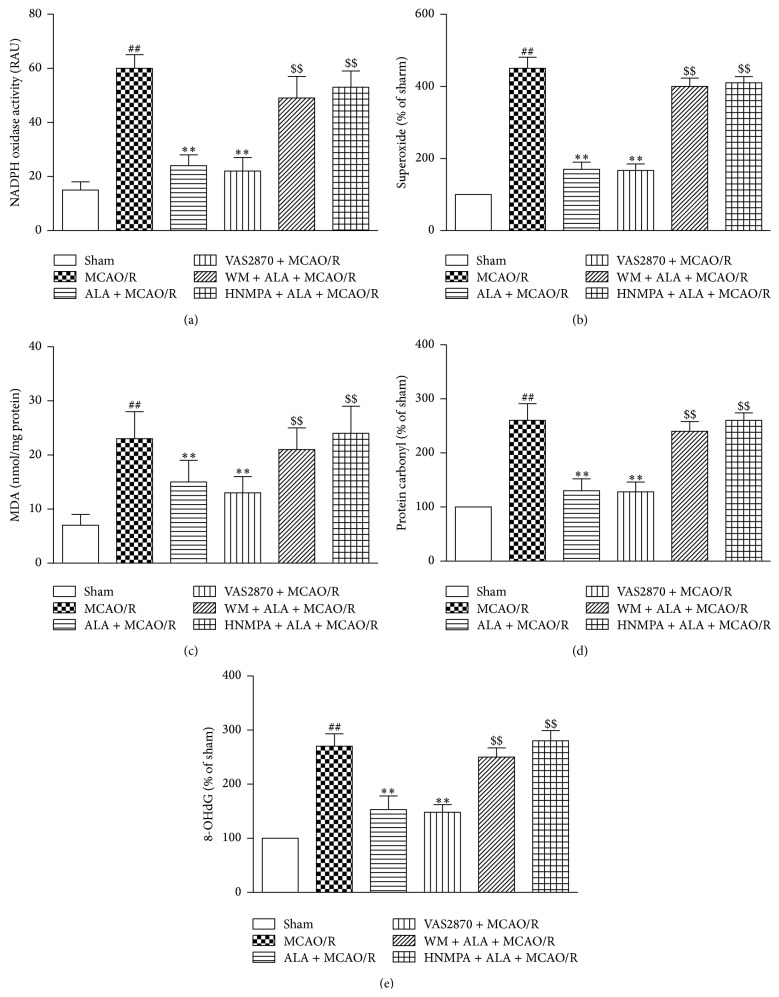
ALA suppressed MCAO/R-induced oxidative stress. (a) ALA inhibited MCAO/R-induced upregulation of NADPH oxidase activity. (b) ALA suppressed MCAO/R-induced superoxide generation and the increment of oxidative stress indicators including MDA (c), protein carbonyl (d), and 8-OHdG (e). For the ALA + MCAO/R group, ALA (40 mg/kg, i.p.) was daily administered to rats for 3 days. For the sham and MCAO/R groups, the rats were given the same amount of ethanol. Data were expressed as means ± SEM. ^##^ *P* < 0.01 compared with the sham group. ^*∗∗*^ *P* < 0.01 compared with the MCAO/R group. ^$$^ *P* < 0.01 compared with the ALA + MCAO/R group.

A noticeable increment of NADPH oxidase activity and a remarkable augment of superoxide production were observed in the cortex of MCAO/R-treated rats (Figures 4(a) and 4(b), *P* < 0.01). In accordance with the increased NADPH oxidase-derived superoxide production, MCAO/R resulted in a significant increase in the levels of MDA, protein carbonyl, and 8-OHdG (Figures 4(c), 4(d), and 4(e), *P* < 0.01). In contrast, ALA could effectively suppress MCAO/R-induced oxidative stress in the cortex of rats (Figure 4, *P* < 0.01). However, all these effects mediated by ALA could be abrogated by coadministration with NADPH oxidase inhibitor VAS2870 (2 mg/kg, i.p.) or PI3K specific inhibitor wortmannin (WM, 0.3 mg/kg, i.p.) or insulin receptor inhibitor HNMPA-(AM)_3_ (5 mg/kg, i.p.) for 3 days (Figure 4, *P* < 0.01).

## 4. Discussion

In the present study, we reported the neuroprotective effects of ALA against CIR injury. We found that administration of ALA (40 mg/kg, i.p.) for 3 days significantly ameliorated brain injury induced by MCAO/R in rats. In accordance with previous studies [14, 15], MCAO/R-induced neurological deficits were effectively attenuated by ALA, as determined by grip test and Rota-rod test. Furthermore, ALA could markedly reduce MCAO/R-induced infarct volume and neuronal apoptosis. However, the molecular mechanism whereby ALA exerts its neuroprotection is largely unknown.

Oxidative stress induced by excessive production of ROS plays a crucial role in the pathogenesis of CIR injury [22]. Excessive ROS directly oxidize several biomacromolecules such as lipids, proteins, and DNA and indirectly activate signaling pathways, resulting in cellular dysfunction and cell apoptosis [23]. Thus, suppressing ROS production may be a novel strategy for neuroprotection against CIR injury. In this study, ALA could inhibit MCAO/R-induced superoxide generation. Subsequently, the elevated levels of oxidative stress indicators including MDA, protein carbonyl, and 8-OHdG induced by MCAO/R were suppressed by ALA. Nevertheless, the underlying mechanism by which ALA suppresses MCAO/R-induced oxidative stress merits deeper investigation.

Multiple studies have proved that NADPH oxidase is the major source of ROS during ischemic stroke [24, 25]. To implore the mechanism underlying the neuroprotective effects of ALA, we focused on NADPH oxidase. In this study, ALA significantly inhibited MCAO/R-induced upregulation of p47-phox and gp91-phox. At the same time, MCAO/R-induced increased NADPH oxidase activity was also abolished by ALA. Importantly, ALA-mediated neuroprotection against oxidative stress was completely abolished by VAS2870. These results suggested that inhibiting NADPH oxidase might play a pivotal role in ALA-mediated neuroprotection against CIR injury. However, the molecular mechanism by which ALA inhibits NADPH oxidase is largely unknown.

Since ALA has been reported to directly modify insulin receptor [26], the possibility that ALA activates the insulin receptor in CIR injury was evaluated. As expected, ALA elicited a pronounced phosphorylation of insulin receptor, suggesting that ALA could activate insulin receptor. Considering that PI3K/Akt signaling pathway is a prototypical event downstream of insulin receptor, whether ALA could activate PI3K/Akt pathway was further evaluated. The phosphorylated Akt was significantly increased in ALA-treated mice, which indicated that ALA could activate PI3K/Akt pathway. Importantly, ALA-mediated PI3K/Akt activation was abrogated by an insulin receptor specific inhibitor HNMPA-(AM)_3_ or a PI3K/Akt specific inhibitor wortmannin. These results suggested that ALA induced insulin receptor-dependent activation of PI3K/Akt signaling pathways. Interestingly, the ALA-mediated inhibition of NADPH oxidase and suppression of oxidative stress were also abolished by HNMPA-(AM)_3_ or wortmannin, suggesting that activating insulin receptor and PI3K/Akt signaling pathways by ALA results in NADPH oxidase inhibition. Nevertheless, the critical components downstream of PI3K/Akt through which ALA inhibits NADPH oxidase remain unclear.

In conclusion, ALA provides neuroprotective effects against MCAO/R-induced brain injury. The mechanism underlying this neuroprotection involves insulin receptor and PI3K/Akt-dependent inhibition of NADPH oxidase.

## Supplementary Material

No significant intergroup difference was noted in the physiologic parameters including cranial temperature, rectal temperature, MABP, glucose, pH, PO_2_, and PCO_2_ and no significant difference was observed in rCBF between MCAO/R and ALA + MCAO/R groups before occlusion, during 5 occlusion, and after reperfusion.
