# Supplementary material for: Alpha-Lipoic Acid Attenuates Cerebral Ischemia and Reperfusion Injury via Insulin Receptor and PI3K/Akt-Dependent Inhibition of NADPH Oxidase
Source: Int J Endocrinol. 2015 Jul 29;2015:903186. doi: 10.1155/2015/903186 (PMC4532939; doi:10.1155/2015/903186)
Supplement: Supplementary file 1 — No significant intergroup difference was noted in the physiologic parameters including cranial temperature, rectal temperature, MABP, glucose, pH, PO2, and PCO2 and no significant difference was observed in rCBF between MCAO/R and ALA + MCAO/R groups before occlusion, during 5 occlusion, and after reperfusion. [file 903186.f1.docx]

**Supplementary materials**

Table.1 Physiological variables in rats

|  | rCBF  % of baseline | Cranial temperature  °C | rectal temperature  °C | MABP  mmHg | Glucose  mmol/L | PH | PO_2_  mmHg | PCO_2_  mmHg |
| --- | --- | --- | --- | --- | --- | --- | --- | --- |
| 30 min before occlusion |  |  |  |  |  |  |  |  |
| Sham | 98±7 | 37.1±0.1 | 37.4±0.1 | 111±11 | 5.95±0.84 | 7.40±0.02 | 120±22 | 38.2±1.7 |
| MCAO/R | 96±8 | 37.2±0.1 | 37.3±0.1 | 115±15 | 5.90±0.71 | 7.41±0.02 | 123±17 | 37.8±1.8 |
| ALA+MCAO/R | 97±6 | 37.2±0.1 | 37.4±0.1 | 114±12 | 5.48±0.76 | 7.41±0.01 | 120±20 | 38.3±1.9 |
| 30 min of occlusion |  |  |  |  |  |  |  |  |
| Sham  MCAO/R  ALA+MCAO/R | 98±6  14±6##  14±5## | 37.2±0.1  37.0±0.1  37.1±0.1 | 37.3±0.1  37.4±0.1  37.3±0.1 | 114±12  116±11  111±13 | 5.97±0.89  5.86±0.78  5.57±0.56 | 7.41±0.01  7.40±0.02  7.41±0.01 | 124±16  125±17  124±10 | 38.1±1.8  37.9±1.7  37.8±1.5 |
| 30min after  reperfusion |  |  |  |  |  |  |  |  |
| Sham  MCAO/R  ALA+MCAO/R | 95±7  96±8  97±8 | 37.1±0.1  37.2±0.1  37.0±0.1 | 37.4±0.1  37.3±0.1  37.4±0.1 | 113±5  112±6  114±10 | 5.83±0.59  5.88±0.67  5.48±0.72 | 7.40±0.01  7.42±0.02  7.41±0.02 | 124±16  125±17  123±14 | 38.0±1.9  37.9±1.6  38.2±1.7 |

Data were expressed as means ±SEM. ## *P* < 0.01 versus the sham group.

**Supplementary Table. 1 Physiological parameters**

No significant changes in rCBF were observed between MCAO/R and ALA+MCAO/R groups after occlusion. No intergroup differences were noted in cranial temperature, rectal temperature, MABP, glucose, pH, PO_2_, and PCO_2_ were observed in the experimental groups before and during occlusion, and after reperfusion (*P* > 0.05). For the ALA+MCAO/R group, ALA (40 mg/kg, i.p.) was daily administered to rats for 3 days. For the sham and MCAO/R groups, the rats were given the same amount of ethanol. Data were expressed as means ± SEM. ##, *P* < 0.01 compared with the sham group.


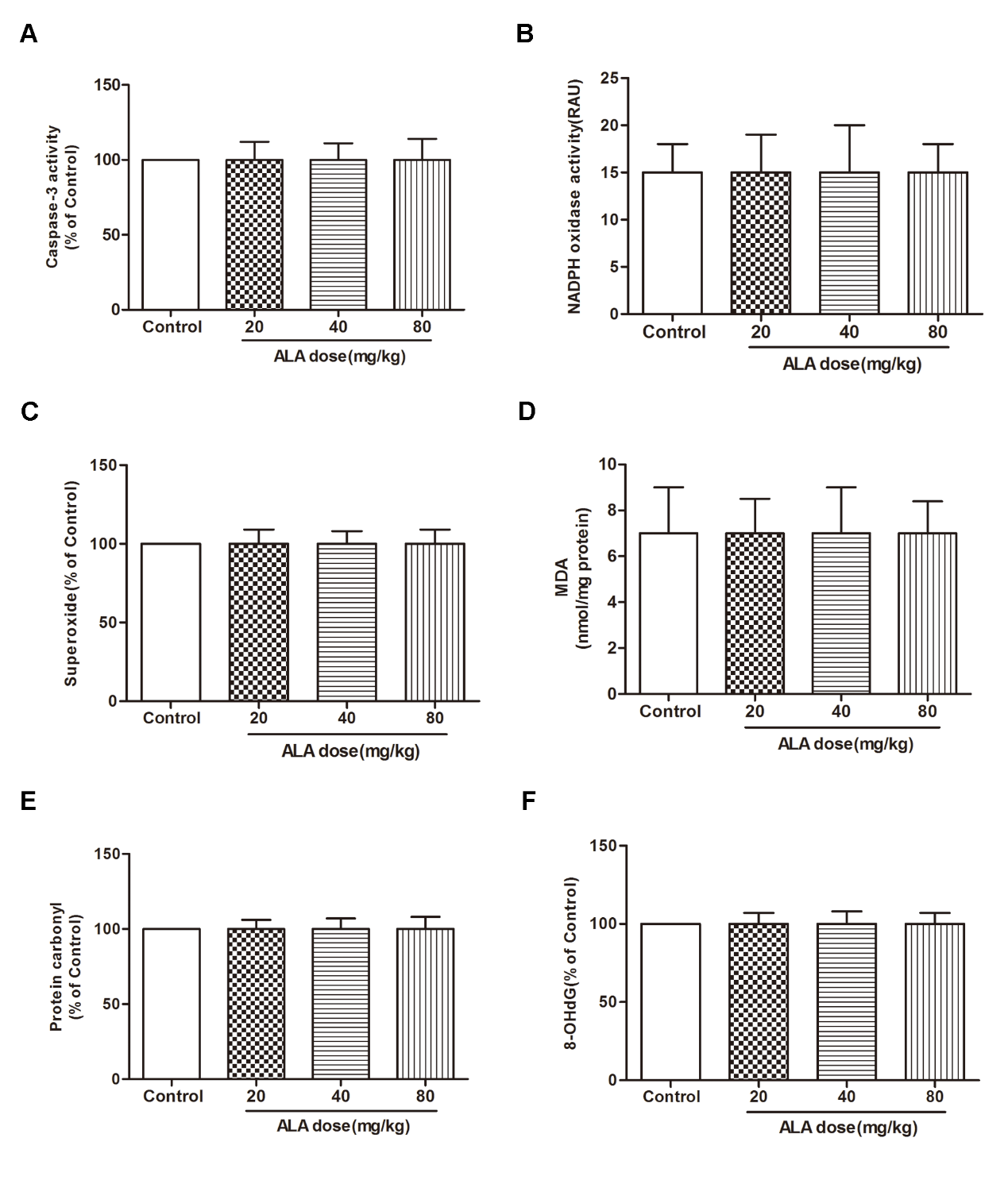


**Supplementary Fig. 1 ALA has no cytotoxicity in rats.**

ALA (20, 40 and 80 mg/kg, i.p.) did not affect the caspase-3 activity (A), NADPH oxidase activity (B), NADPH oxidase-derived superoxide production (C), the levels of MDA (D), protein carbonyl (E) and 8-OHdG (F) in cortex of rats. For the ALA groups, ALA (20, 40 and 80 mg/kg, i.p.) was daily administered to rats for 3 days. For the control group, the rats were given the same amount of ethanol.

**
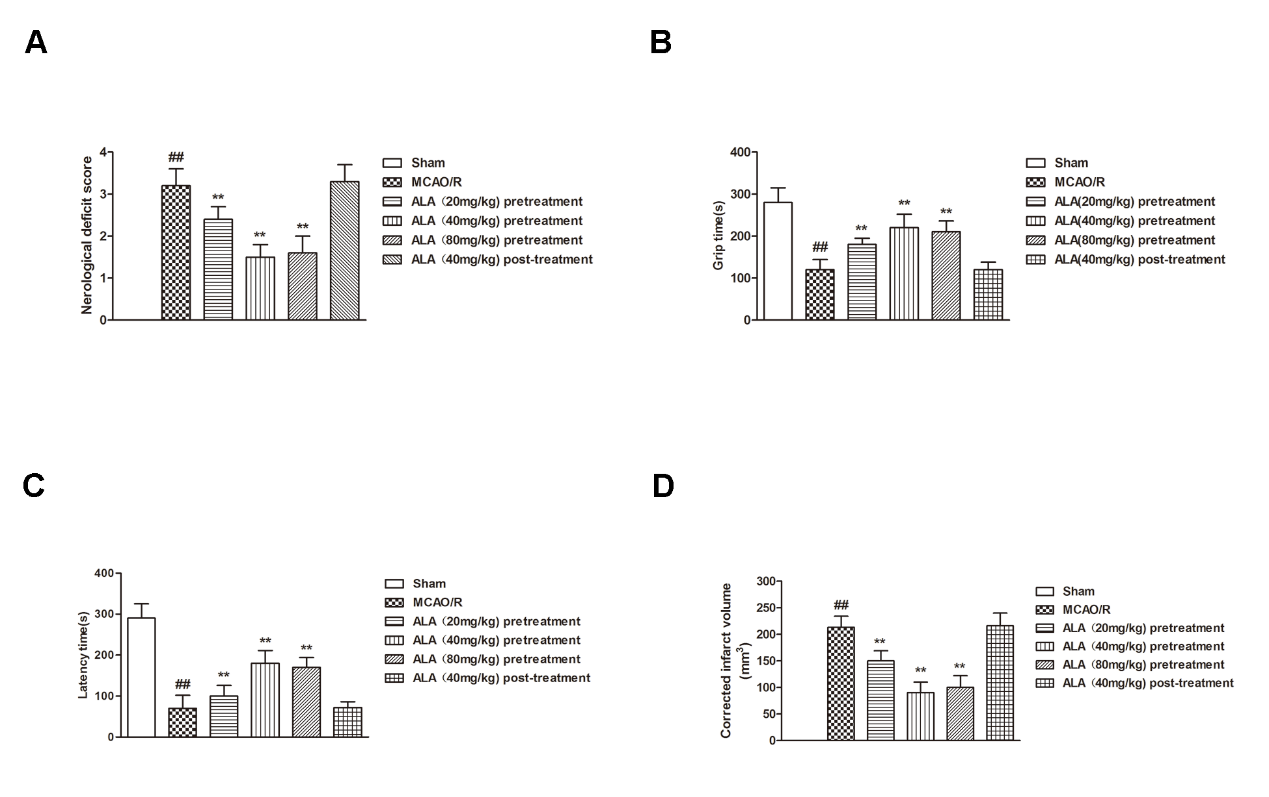
**

**Supplementary Fig. 2 Dose selection**

Pretreatment of rats with ALA (40 mg/kg) provided maximum neuroprotection against neurological deficits (A), the reduced grip time (B), the decreased latency time in Morris water time (C), and the increased cerebral infarction volumes (D) induced by MCAO/R. For the ALA+MCAO/R group, ALA (20, 40 and 80 mg/kg, i.p.) was daily administered to rats for 3 days. For the sham and MCAO/R groups, the rats were given the same amount of ethanol. Data were expressed as means ± SEM. ##, *P* < 0.01 compared with the sham group. **, *P* < 0.01 compared with the MCAO/R group.
